# Supplementary material for: HIV-1 Gag targeting to the plasma membrane reorganizes sphingomyelin-rich and cholesterol-rich lipid domains
Source: Nat Commun. 2023 Nov 21;14:7353. doi: 10.1038/s41467-023-42994-w (PMC10663554; doi:10.1038/s41467-023-42994-w)
Supplement: Supplementary file 1 — Supplementary Information [file 41467_2023_42994_MOESM1_ESM.pdf]

Supplementary information for

HIV-1 Gag targeting to the plasma membrane reorganizes sphingomyelin- and cholesterol-rich lipid domains

Nario Tomishige[1,2]*, Maaz Bin Nasim[1], Motohide Murate[1,2], Brigitte Pollet[1], Pascal Didier[1], Julien Godet[1], Ludovic Richert[1], Yasushi Sako[2], Yves Mély[1]* and Toshihide Kobayashi[1,2]*

*Corresponding author. Email: nario.tomishige@unistra.fr (N.T.); yves.mely@unistra.fr (Y.M.); toshihide.kobayashi@unistra.fr (T.K.)

This PDF file includes:

Supplementary Figures
Supplementary Fig. 1 to 17

Supplementary Tables
Supplementary Table 1 to 6

Supplementary Note
Validation of the double exponential equation model in the analysis of FLIM-FRET data.

Localization uncertainties of fluorescent probes used in the super-resolution microscopy.

Kernel density estimate (KDE) used in flimDiagRam.

Supplementary reference

Unprocessed images of all TLC plates and blots

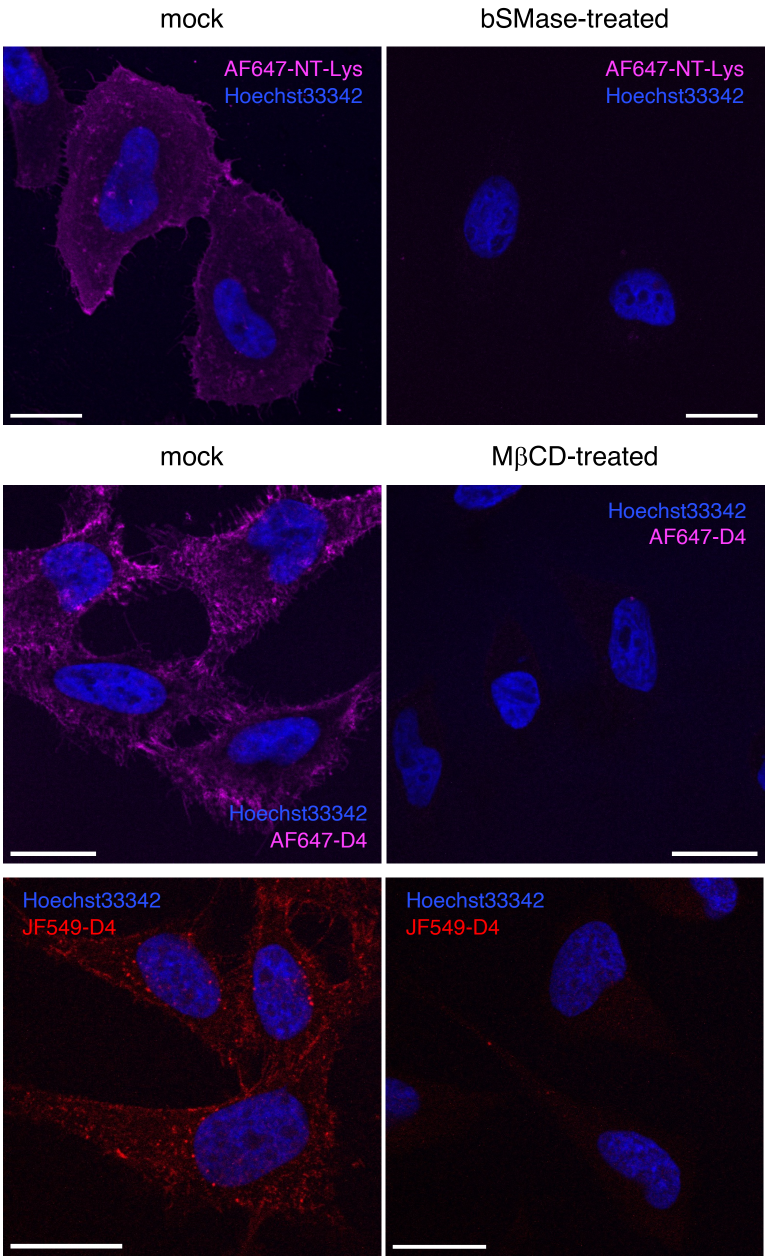

Supplementary Fig. 1 Binding specificity of lipid probes newly developed in this study
HeLa cells were labeled with 0.2 μM AF647-NT-Lys, 0.8 μM AF647-D4, or 0.4 μM JF549-D4 after treatment with mock and either 0.125 U bacterial SMase (bSMase) or 10 mM methyl-β-cyclodextrin (MbCD) at 37°C for 30 min, for NT-Lys or D4 labeling, respectively. The cells were fixed with 4% PFA, and the nucleus was stained with Hoechst33342. The removal of either SM by bSMase treatment or Chol by MbCD abolished AF647-NT-Lys or AF647-D4 and JF549-D4 labeling, indicating the specificity of these probes to the respective lipids. Bar, 20 μm.

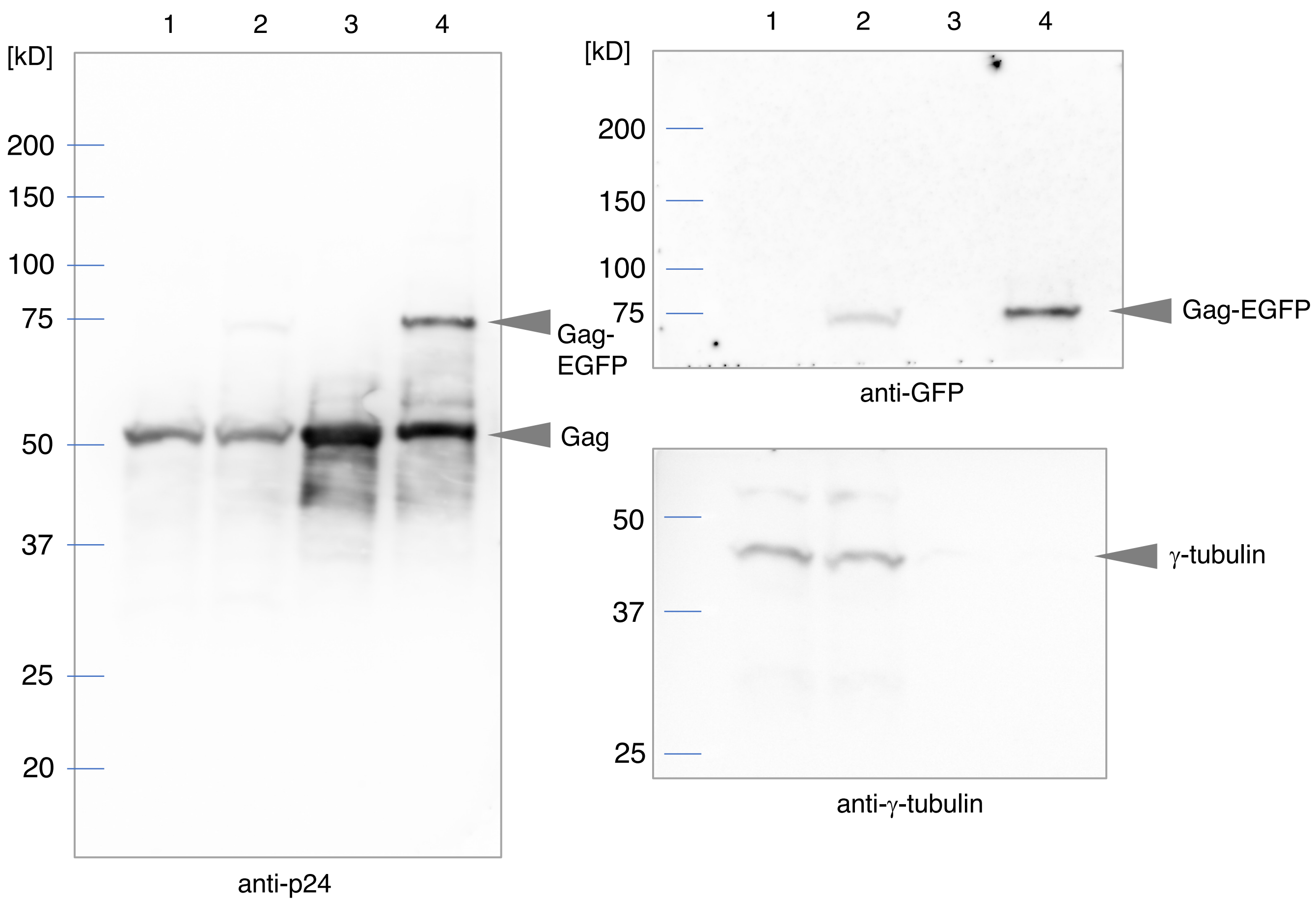

Supplementary Fig. 2 VLP production from 293T cells transfected with a mixture of Gag/Gag-EGFP (3:1). Lysate prepared from 293T cells and VLP fraction prepared from medium supernatant, as described in Supplementary Methods, were subjected to Western blotting using antibodies against p24, GFP, and γ-tubulin. Lane1, lysate from non-tagged Gag-expressing cells; lane 2: lysate from Gag/Gag-EGFP expressing cells; lane 3: VLP fraction from non-tagged Gag-expressing cells; lane 4: VLP fraction from Gag/Gag-EGFP expressing cells. The positions of Gag-EGFP, Gag, and γ-tubulin were indicated with arrowheads. Representative images in two independent experiments were shown. The positions of molecular weight markers are indicated in the left in [kD]. The figure showed that total amounts (Gag and Gag + Gag-EGFP) of Gag in either lysate or VLP are comparable between samples of cells transfected with Gag alone and Gag/Gag-EGFP.

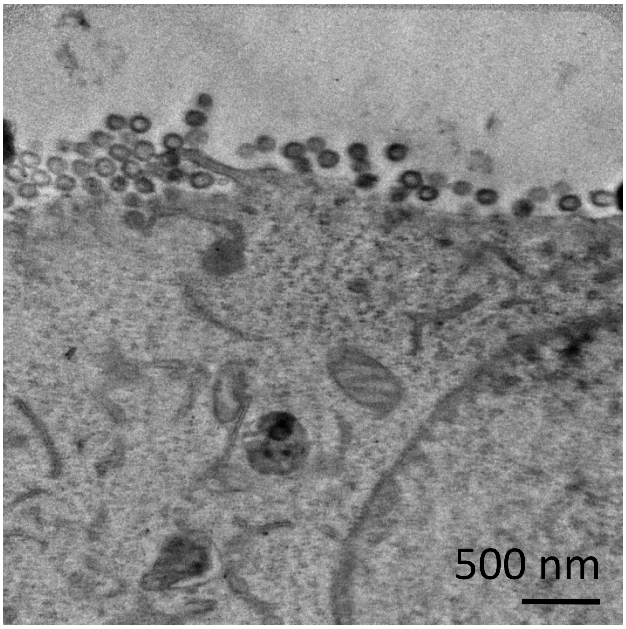

Supplementary Fig. 3 EM pictures of VLP production on the PM by Gag/Gag-EGFP (3:1). The sample was prepared after 24 hrs of transfection of HeLa cells with Gag/Gag-EGFP plasmids, as described in Supplementary Methods.

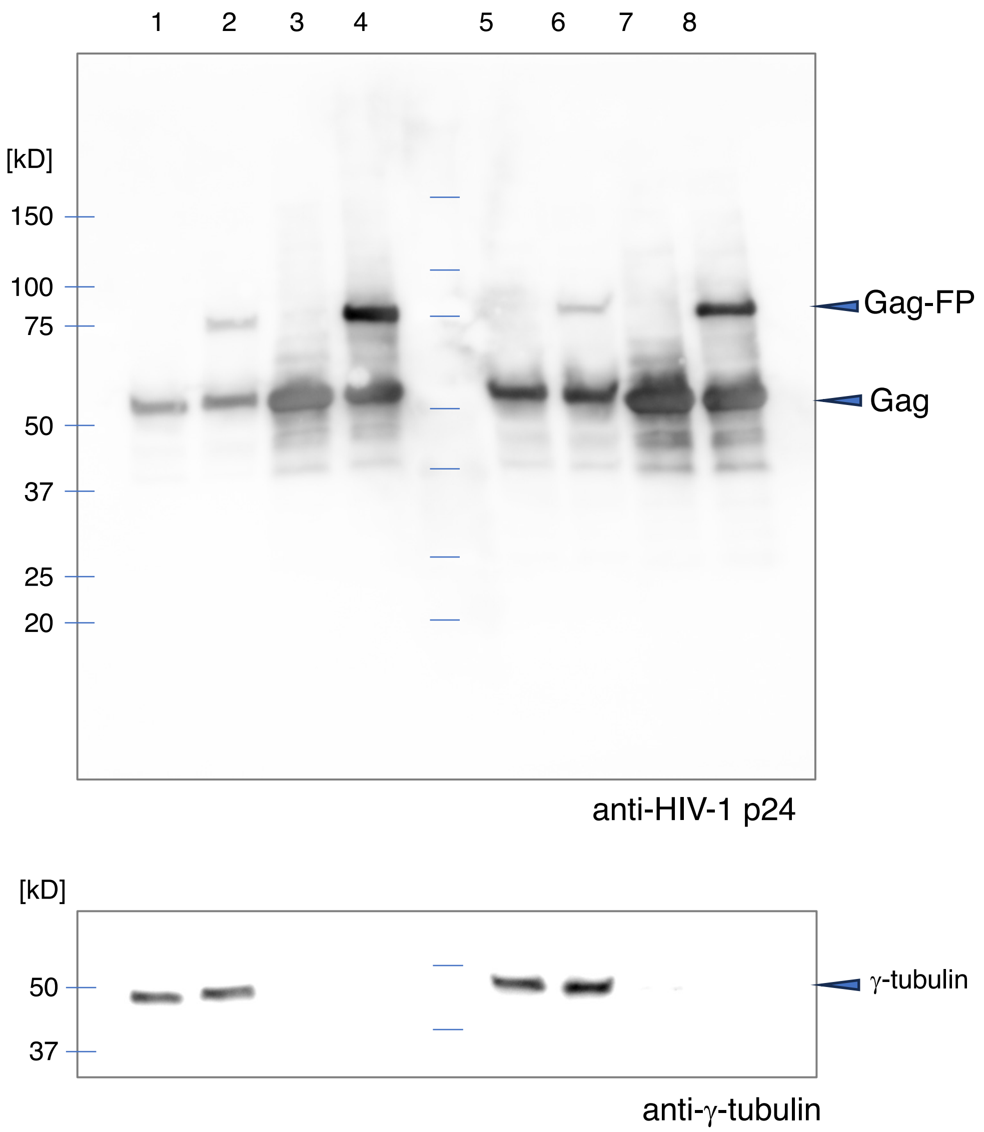

Supplementary Fig. 4 VLP production from 293T cells transfected with a mixture of Gag/Gag-FP (3:1). Lysate prepared from 293T cells and VLP fraction prepared from medium supernatant, as described in Methods, were subjected to Western blotting using antibodies against p24 and γ-tubulin. Lane1and 5, lysate from non-tagged Gag-expressing cells; lane 2: lysate from Gag/Gag-mEos4b expressing cells; lane 3 and 7: VLP fraction from non-tagged Gag-expressing cells; lane 4: VLP fraction from Gag/Gag-mEos4b expressing cells; lane 6: lysate from Gag/Gag-mTagBFP2 expressing cells; lane 8: VLP fraction from Gag/Gag-mTagBFP2 expressing cells. The positions of Gag-FP, Gag, and γ-tubulin were indicated with arrowheads. A representative image was shown. The positions of molecular weight markers are indicated in the left in [kD]. The figure showed that total amounts (Gag and Gag + Gag-FP) of Gag in either lysate or VLP are comparable between samples of cells transfected with Gag alone and Gag/Gag-FP.

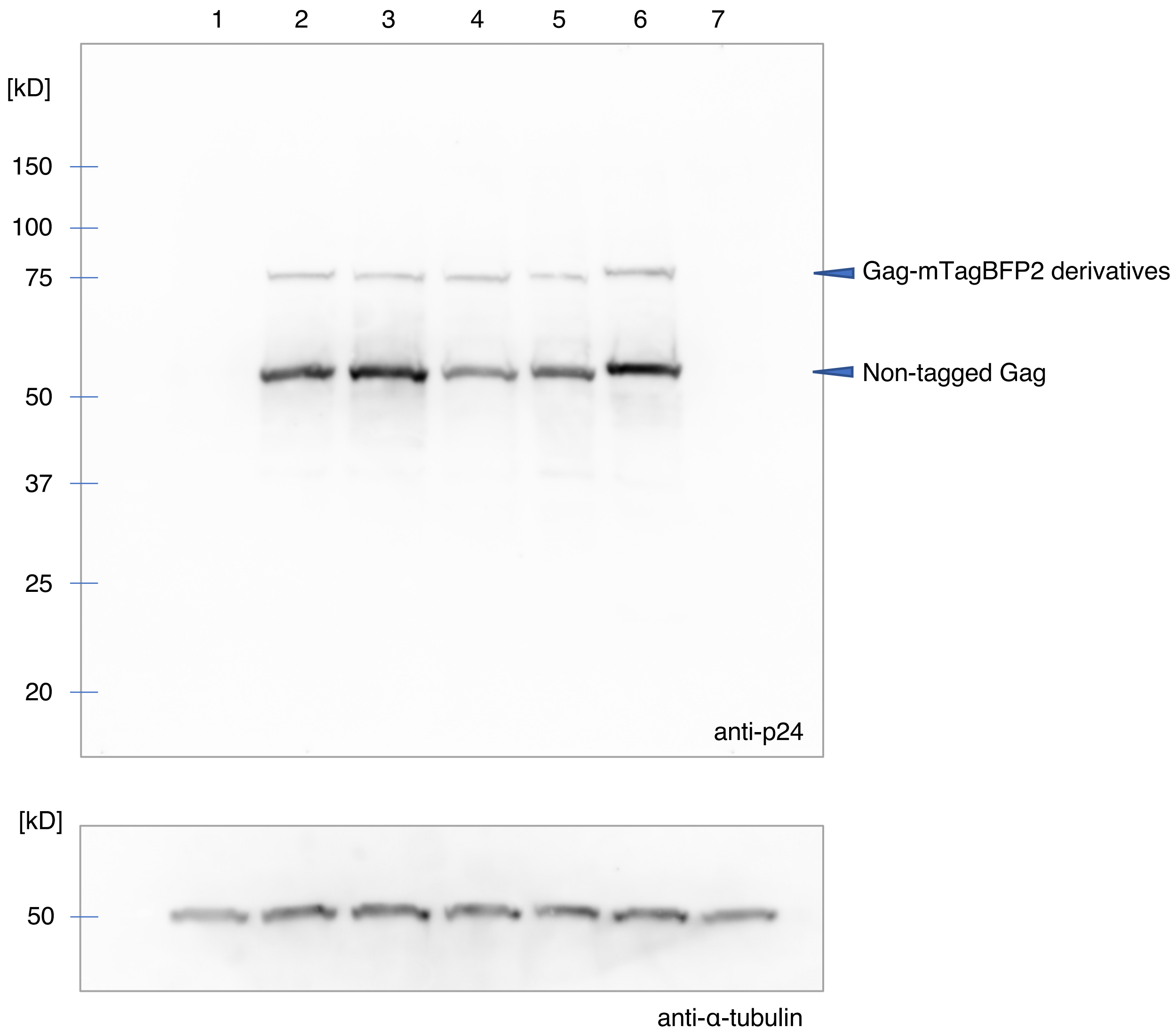

Supplementary Fig. 5 Characterization of Gag-mTagBFP2 derivatives expression in cells. HeLa cells were transfected with a mixture of Gag/Gag-mTagBFP2 derivatives (3:1). Cell lysate was prepared 24 h after transfection. The proteins expressed in the cell lysate were examined by Western blotting using antibodies against p24 and α-tubulin. Representative images in two independent experiments were shown. The positions of these proteins and protein size markers were indicated on the right and left, respectively. Lane 1, lysate prepared from cells transfected with the empty vectors; lane 2, Gag/Gag-mTagBFP2; lane 3, Gag-ΔL/Gag-ΔL-mTagBFP2; lane 4, Gag-P99A/Gag-P99A-mTagBFP2; lane 5, Gag-EE/Gag-EE-mTagBFP2; lane 6, Gag-WM/Gag-WM-mTagBFP2; lane 7, mock-transfected.

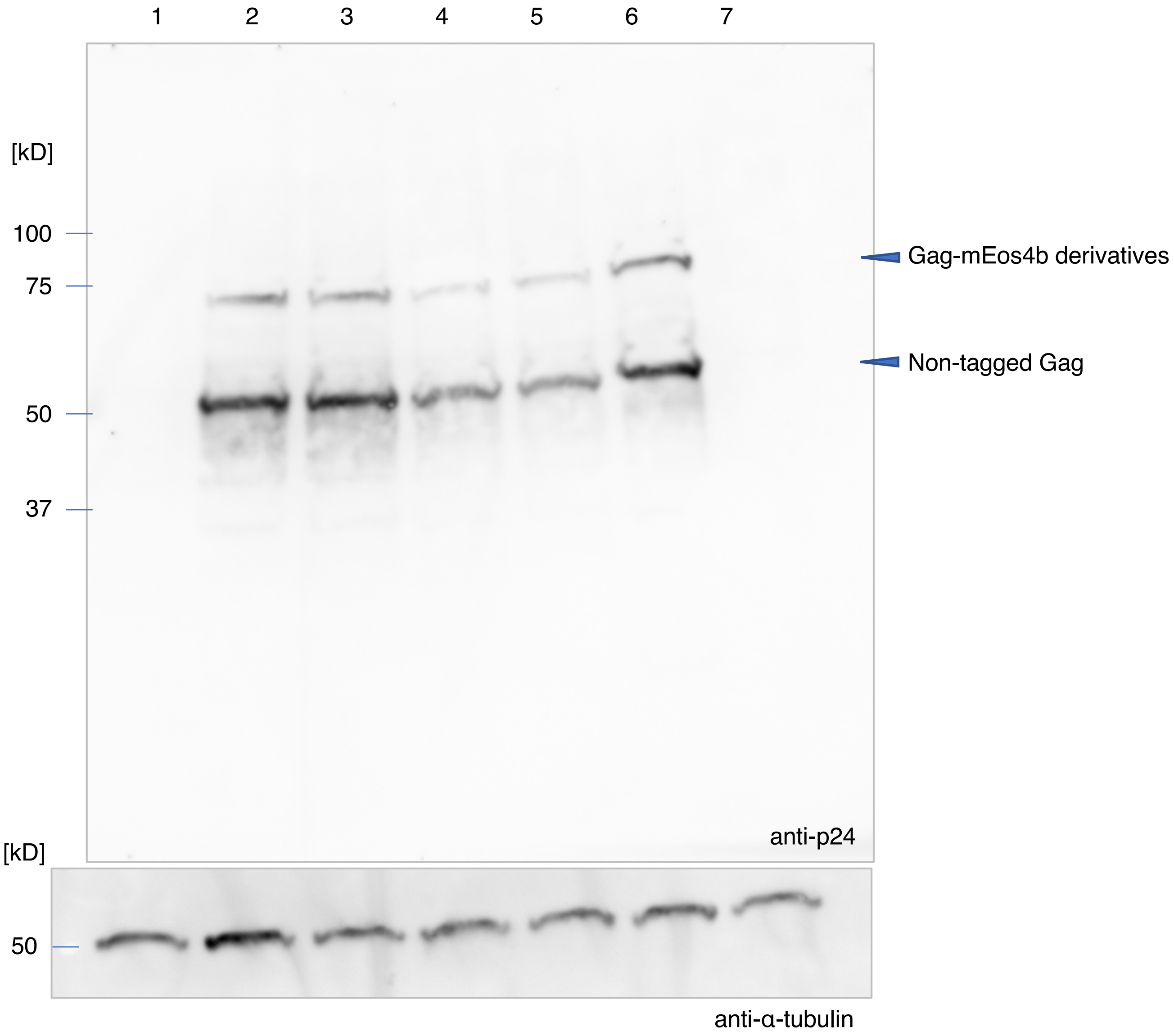

Supplementary Fig. 6 Characterization of Gag-mEos4b derivatives expression in cells. HeLa cells were transfected with a mixture of Gag/Gag-mEos4b derivatives (3:1). Cell lysate was prepared 24 h after transfection. The proteins expressed in the cell lysate were examined by Western blotting using antibodies against p24 and α-tubulin. The positions of these proteins and molecular weight markers were indicated on the right and left of the panels, respectively. Representative images in two independent experiments were shown. Lane 1, lysate prepared from cells transfected with the empty vector; lane 2, Gag/Gag-mEos4b; lane 3, Gag-ΔL/Gag-ΔL-mEos4b; lane 4, Gag-P99A/Gag-P99A-mEos4b; lane 5, Gag-EE/Gag-EE-mEos4b; lane 6, Gag-WM/Gag-WM-mEos4b; lane 7, mock-transfected.

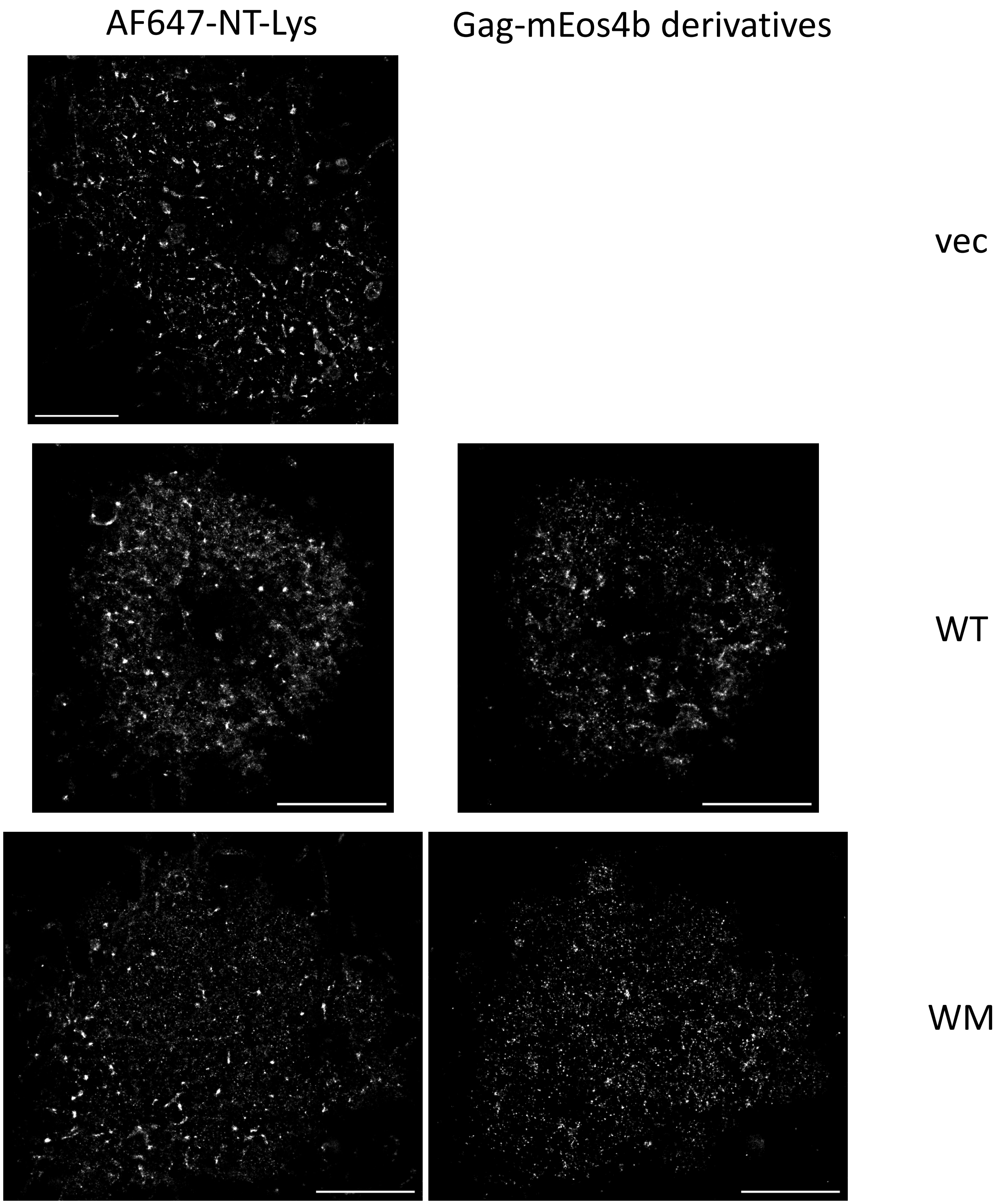

Supplementary Fig. 7 Whole reconstructed images of a single cell in PALM/dSTORM imaging. From top, a representative image of AF647-NT-Lys in a vector-transfected cell labeled with AF647-NT-Lys (vec), images of AF647-NT-Lys and Gag WT-mEos4b in a Gag WT/Gag WT-mEos4b-expressing cell labeled with AF647-NT-Lys (WT), and images of AF647-NT-Lys and Gag-WM-mEos4b in a Gag-WM/Gag-WM-mEos4b-expressing cell labeled with AF647-NT-Lys (WM). Bar, 5 μm.

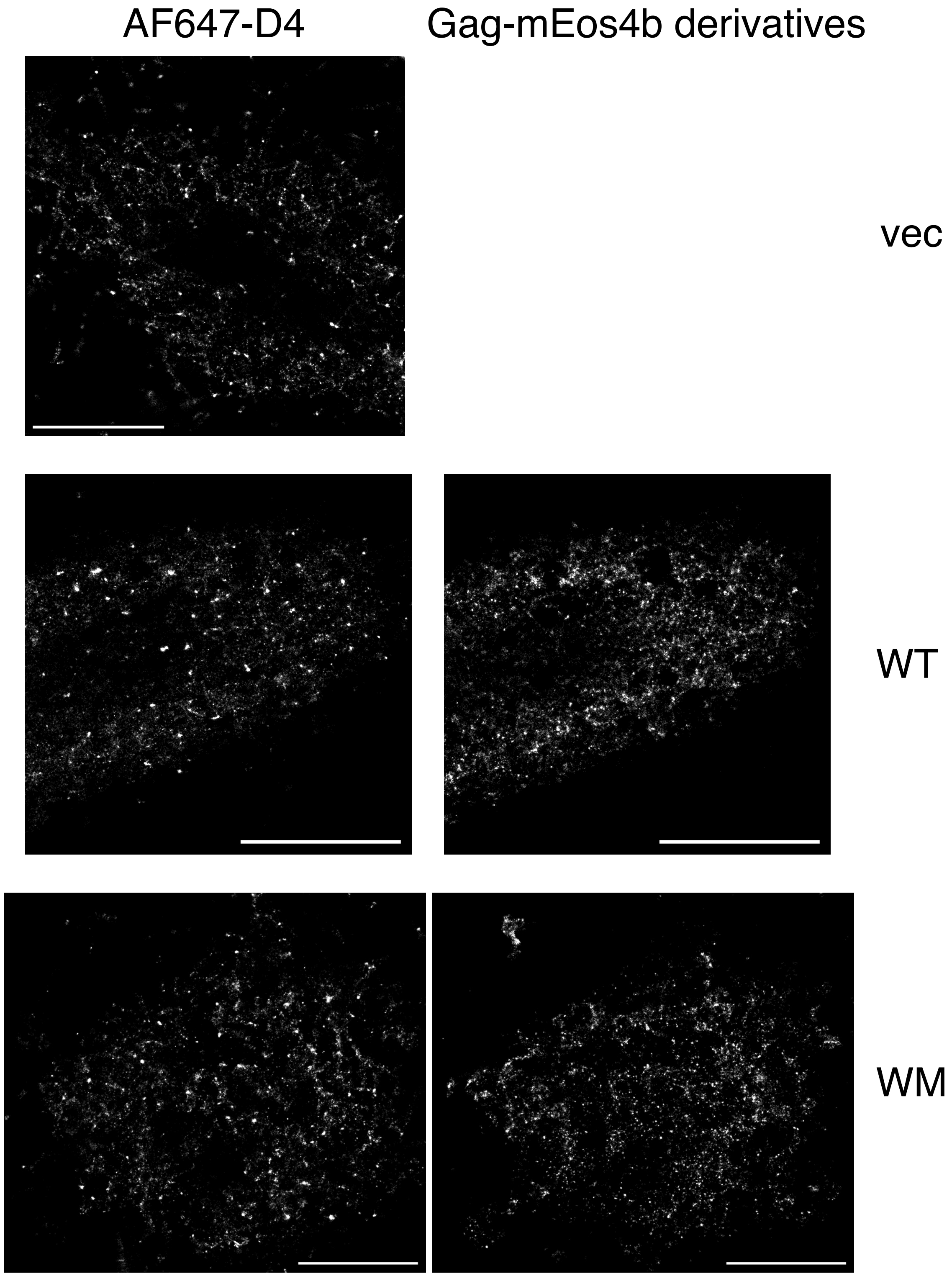

Supplementary Fig. 8 Whole reconstructed images of a single cell in PALM/dSTORM imaging. From the top, a representative image of AF647-D4 in a vector-transfected cell labeled with AF647-D4 alone (vec), images of AF647-D4 and Gag WT-mEos4b in a Gag WT/Gag WT-mEos4b-expressing cell labeled with AF647-D4 (WT), and images of AF647-D4 and Gag-WM-mEos4b in a Gag-WM/Gag-WM-mEos4b-expressing cell labeled with AF647-D4 (WM). Bar, 5 μm.

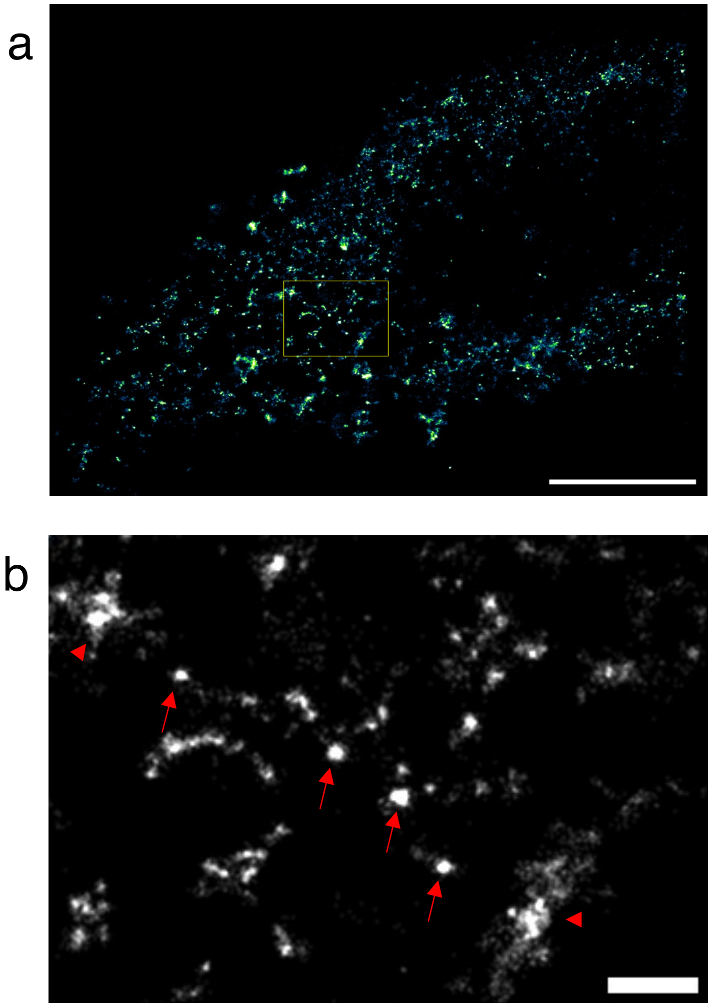

Supplementary Fig. 9 Estimation of Gag WT-mEos4b domain diameter in PALM/dSTORM imaging. a a representative image of a Gag WT-mEos4b-expressing cell. Bar, 5 μm. **b** the zoomed image in yellow ROI in (**a**). The arrows and arrowheads indicated round shaped domains and interconnected large domains including the round shaped domains. Bar, 500 nm.

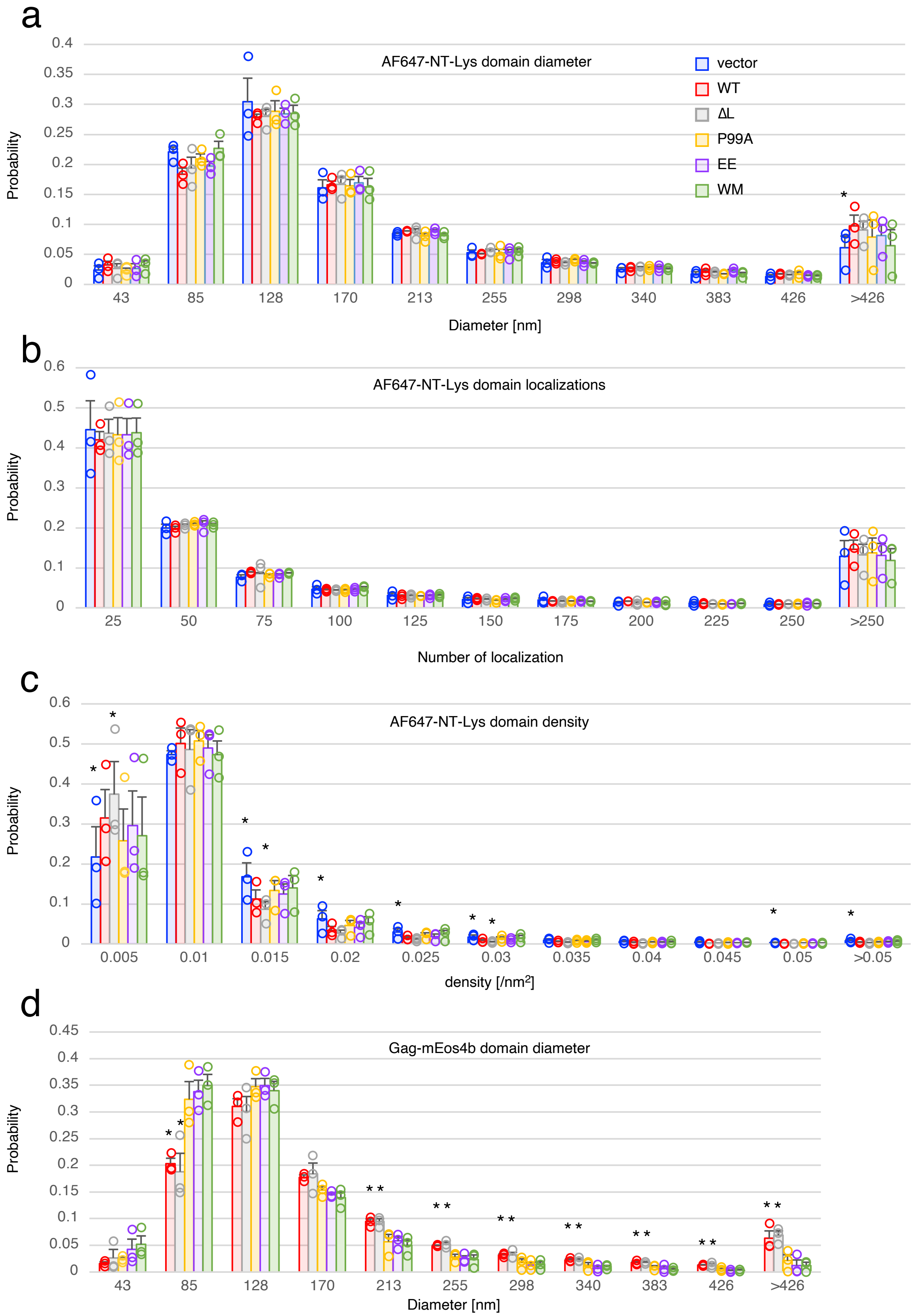

Supplementary Fig. 10 Parameter distribution of individual AF647-NT-Lys and Gag-mEos4b domains. a-d Probability distributions of domain diameter (**a**), number of localizations (**b**), and density (**c**) of AF647-NT-Lys domains and domain diameter of Gag-mEos4b domains (**d**). The numbers on the x- and y-axis indicate the upper limit of each bin and probability, respectively. From the left, blue, red, gray, yellow, purple, and green bars indicate the means ± SEM in cells transfected with vectors, Gag WT, -ΔL, -P99A, -EE, and -WM, respectively, in three independent experiments (n = 17, 18, 17, 16, 16, and 16 for vec, WT, ΔL, P99A, EE, and WM in each). Circles in each color indicates values in the three experiments. * indicates a bar showing any differences with the others in one-way ANOVA post hoc Tukey test. The detailed statisticss are shown in **Supplementary Table 1-3**.

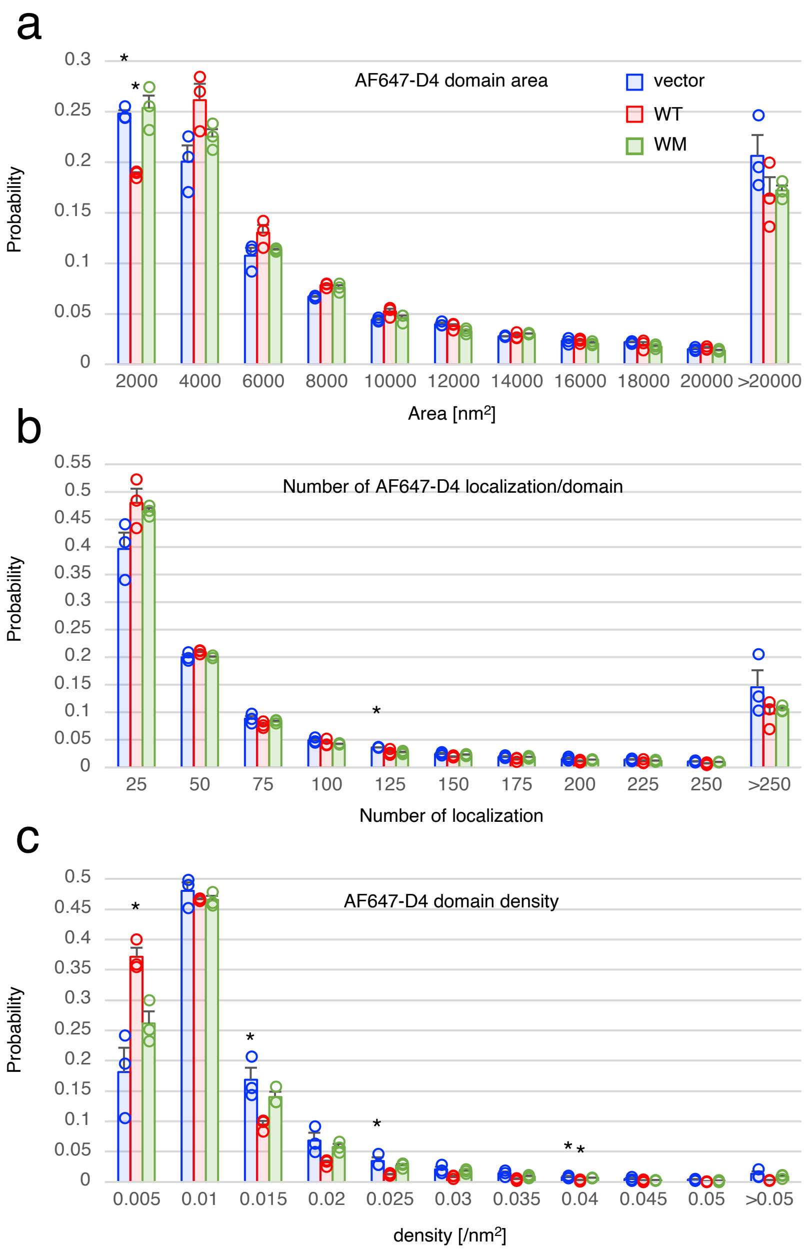

Supplementary Fig. 11 Parameter distribution of individual AF647-D4 domains. a-c Probability distributions of domain diameter (**a**), number of localizations (**b**), and density (**c**) of AF647-D4 domains. The numbers in the x- and y-axis indicate the upper limit of each bin and probability, respectively. From the left, blue, red, and green bars indicate the means ± SEM in cells transfected with vectors, Gag WT, and -WM, respectively, in three independent experiments (n = 15, 16, and 16 for vec, WT, and WM over three experiments). Circles in each color indicate values in the three experiments. * indicates a bar showing any differences with the others in the one-way ANOVA post-hoc Tukey test (**a**-**c**). The detailed statistics in the ANOVA test are shown in **Supplementary Table 4-6**.

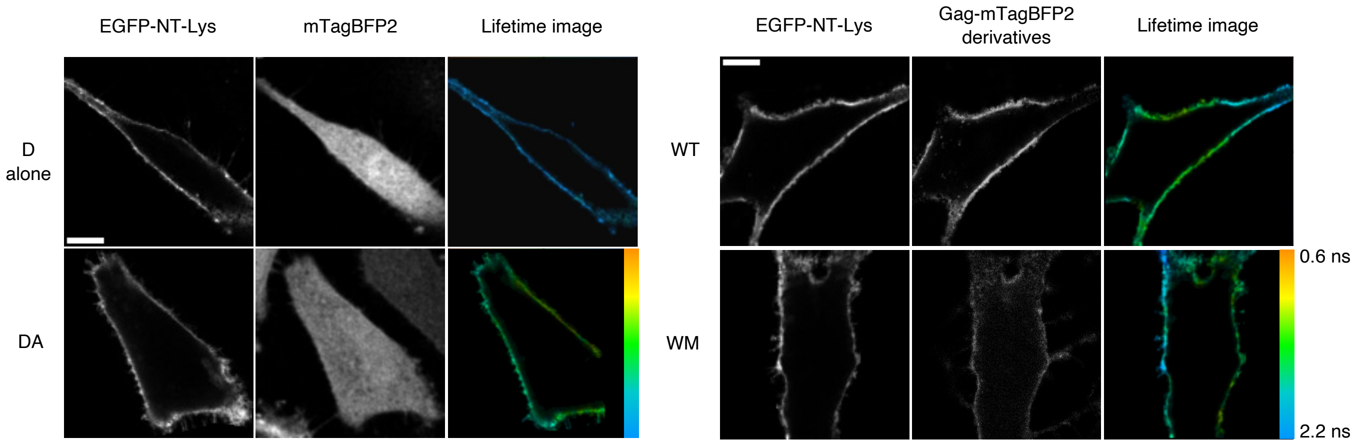

Supplementary Fig. 12 Representative images of EGFP-NT-Lys, mTagBFP2 or Gag-mTagBFP2 derivatives, and lifetime τ_1 related to Fig. 5. D alone indicates mTagBFP2-expressing cells labeled with EGFP-NT-Lys alone. DA, WT, and WM indicate mTagBFP2, Gag WT/Gag WT-mTagBFP2, and Gag-WM/Gag-WM-mTagBFP2 -expressing cells labeled with EGFP-NT-Lys and mCherry-NT-Lys, respectively. Color gauges on the right indicate the lifetime τ_1 corresponding to 0.6 to 2.2 ns. Bar, 10 μm.

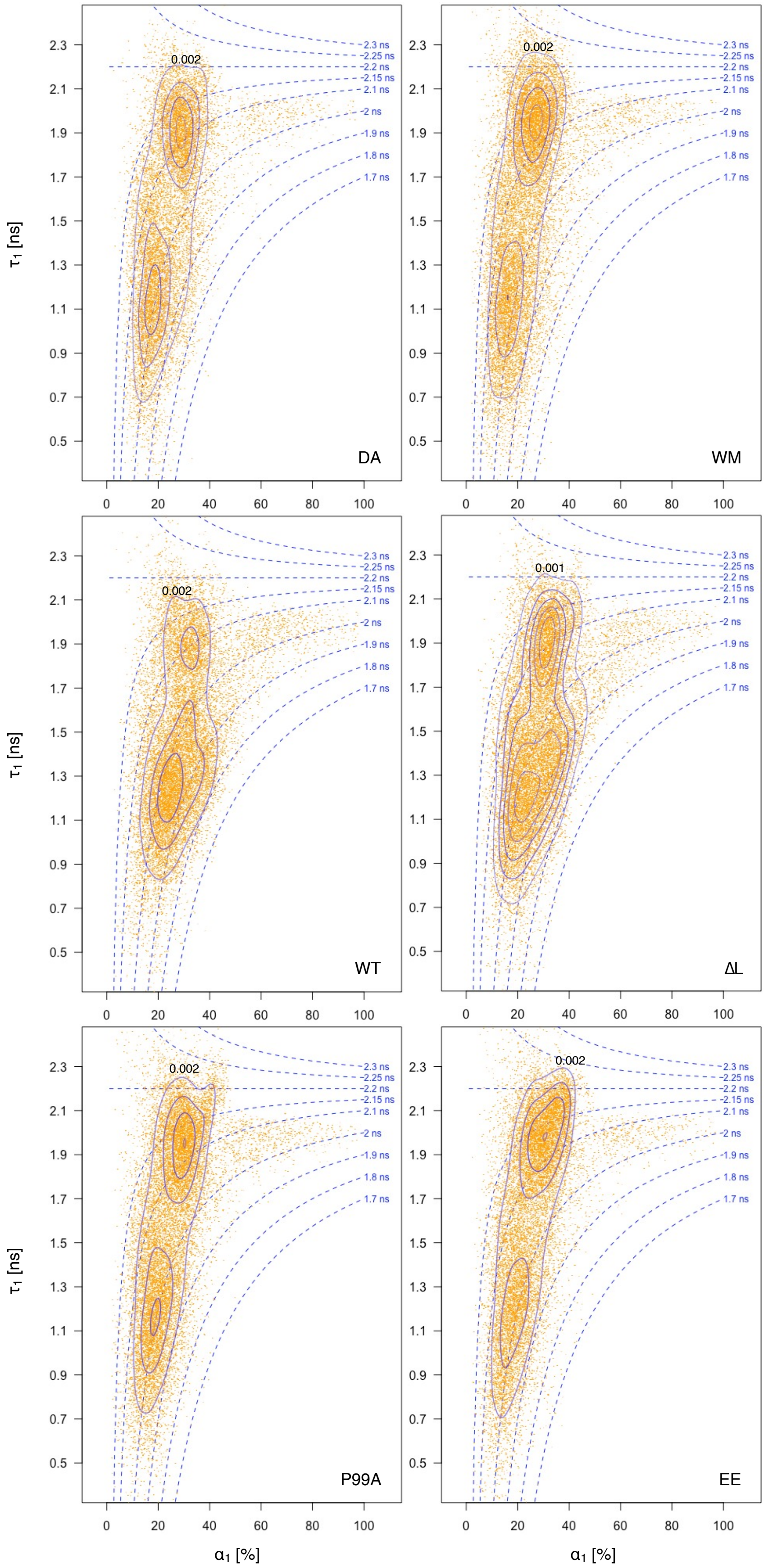

Supplementary Fig. 13 Representative FLIM plot of τ_1 and α_1 distribution related to Fig. 6. The distribution of the interacting population in each pixel was shown as scatter plots according to its lifetime τ_1 [ns] (y-axis) and amplitude α_1 [%] (x-axis). The data combined from all cells (n = 10) in one experiment were shown. DA, WT, P99A, WM, ΔL, and EE indicate cells without and with Gag WT/Gag WT-mTagBFP2, Gag-P99A/Gag-P99A-mTagBFP2, Gag-WM/Gag-WM-mTagBFP2, Gag-ΔL/Gag-ΔL-mTagBFP2, and Gag-EE/Gag-EE-mTagBFP2, respectively. The blue contour lines on the distribution indicate the population density estimated with KDE. The contour lines were drawn every 0.002 from 0.002 of the outermost except for ΔL, in which every 0.001 from 0.001. The blue dotted lines indicate the global lifetimes (indicated in blue) when fixing the lifetime τ_2 of the non-interacting population to 2.2 ns.

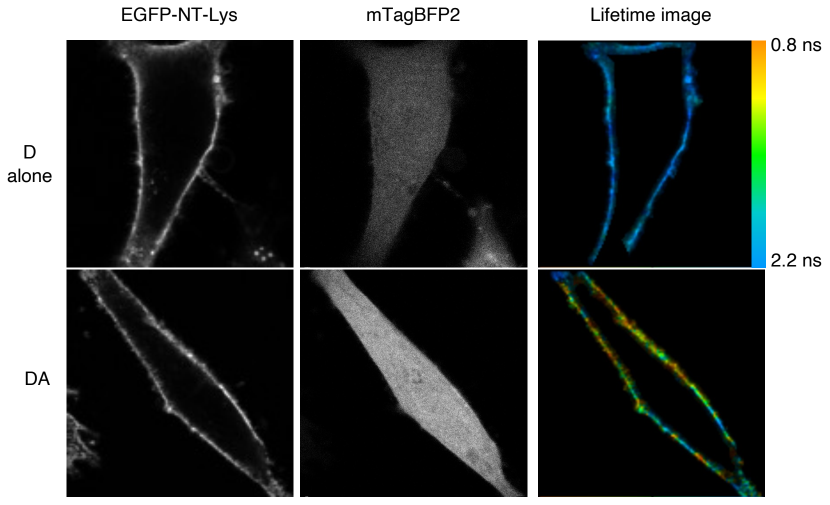

Supplementary Fig. 14 Correlation between lifetime and Gag-mTagBFP2 intensity related to Fig. 6. The representative images of EGFP-NT-Lys, mTagBFP2, and lifetime τ_1 [ns]. D alone indicates mTagBFP2-expressing cells labeled with EGFP-NT-Lys alone. DA indicates mTagBFP2-expressing cells labeled with EGFP-NT-Lys and mCherry-D4. The color gauge on the right indicates the lifetime corresponding to 0.8 to 2.2 ns. Bar, 10 μm.

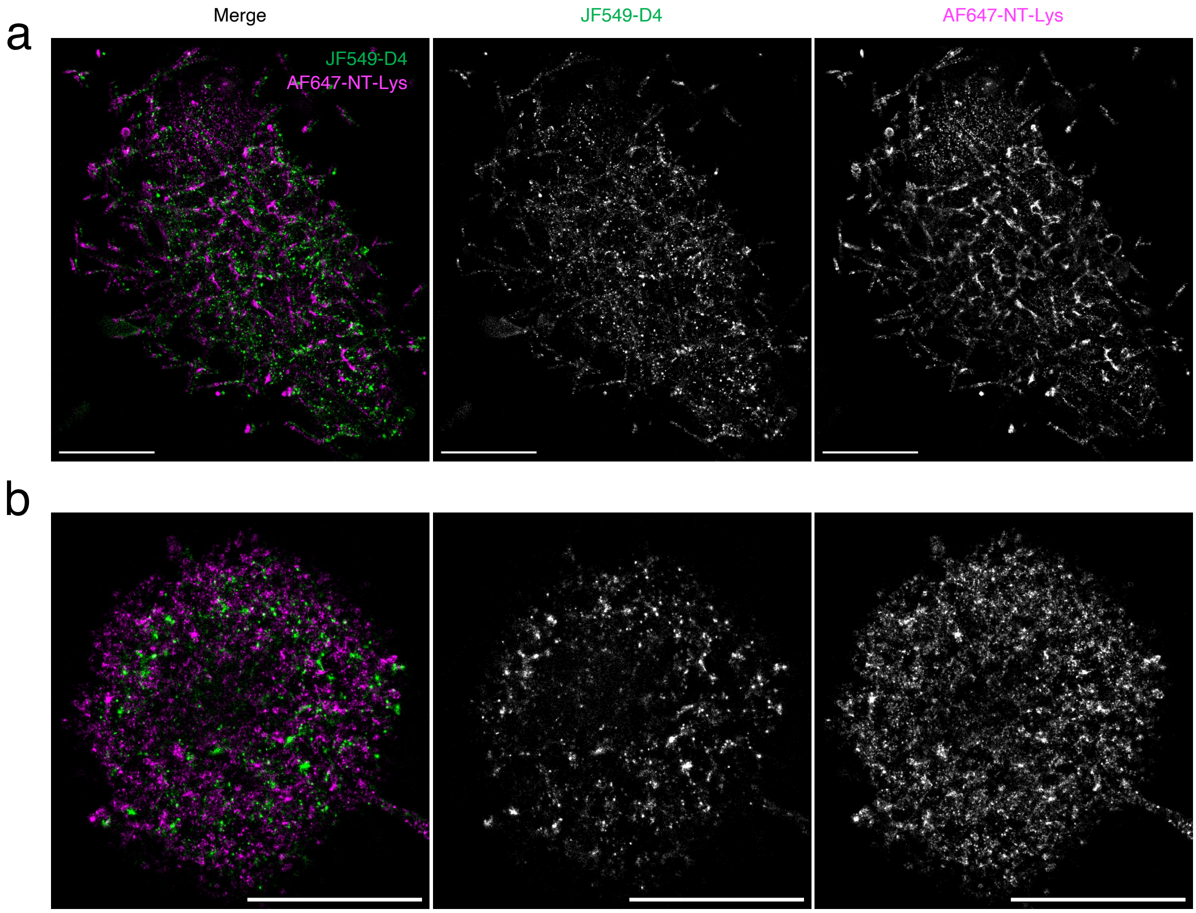

Supplementary Fig. 15 Whole reconstructed images of JF549-D4 and AF647-NT-Lys in two-color dSTORM. a Merged, JF549-D4, and AF647-NT-Lys images of a representative cell labeled with JF549-D4 and AF647-NT-Lys and transfected with a vector. **b** Merged, JF549-D4, and AF647-NT-Lys images of a representative Gag/Gag-EGFP-expressing cell labeled with JF549-D4. Bar, 5 μm.

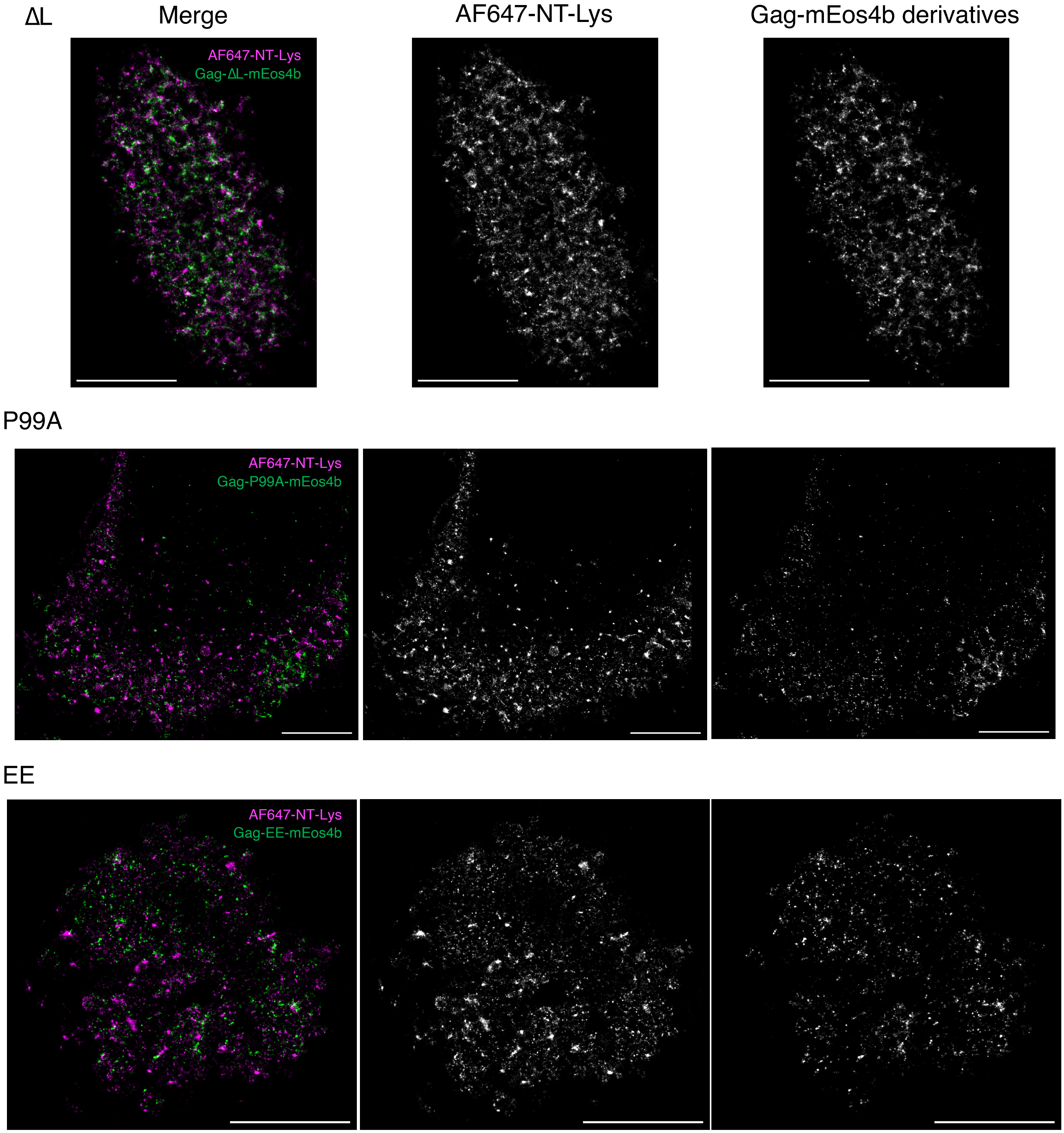

Supplementary Fig. 16 Whole reconstructed images of a Gag-curvature mutant-expressing cell in PALM/dSTORM. From top, Merged, AF647-NT-Lys, and Gag-mEos4b mutant images of a Gag-ΔL/Gag-ΔL-mEos4b (ΔL) -, Gag-P99A/Gag-P99A-mEos4b (P99A) -, and Gag-EE/Gag-EE-mEos4b (EE) - expressing cell labeled with AF647-NT-Lys. Bar, 5 μm.

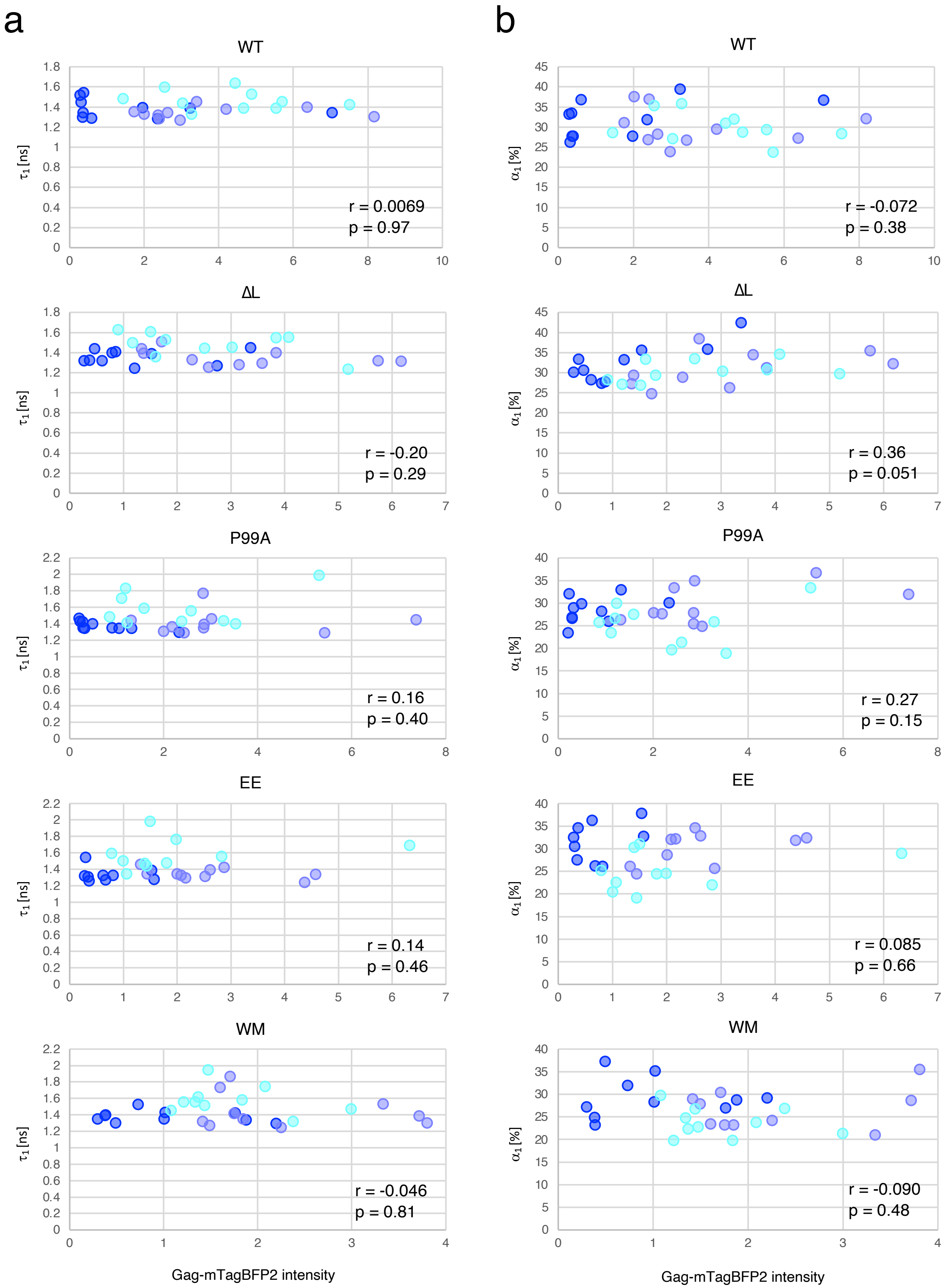

Supplementary Fig. 17 No correlations between Gag-mTagBFP2 intensity and lifetime τ_1 or α_1 among cells. a and **b** Scatter plots of mean lifetime τ_1 (**a**) or mean amplitude α_1 (**b**) and Gag-mTagBFP2 derivatives mean intensities (x-axis, photon number/unit time) in cells expressing indicated Gag/Gag-mTagBFP2 derivatives in FLIM-FRET experiments of EGFP-NT-Lys and mCherry-D4 (in Fig. 6). Colored dot indicates a mean in each cell in three independent experiments (n = 30 for all samples except for EE (n = 29) over the three experiments). Pearson correlation coefficients (r) and p values are shown in the graphs.

Tested combination		Diameter [nm]
		>426
vector	WT	*
	ΔL	ns
	P99A	ns
	EE	ns
	WM	ns
WT	ΔL	ns
	P99A	ns
	EE	ns
	WM	ns
ΔL	P99A	ns
	EE	ns
	WM	ns
P99A	EE	ns
	WM	ns
EE	WM	ns

Supplementary Table 1 Results of one-way ANOVA post-hoc Tukey test for AF647-NT-Lys domain diameter distribution related to Supplementary Fig. 10a. First two columns indicate combinations tested statistically. Third column indicates the results of the test in Supplementary Fig. 10a. * indicates the significant difference at the significance level of 0.05 in one-way ANOVA post-hoc Tukey test. ns indicates not significant. Detailed statistics are shown in the Source Data.

Tested combination		Density [/nm2]						
		0.005	0.015	0.02	0.025	0.03	0.05	>0.05
vector	WT	*	*	ns	ns	*	ns	ns
	ΔL	***	**	*	*	*	**	*
	P99A	ns	ns	ns	ns	ns	ns	ns
	EE	ns	ns	ns	ns	ns	ns	ns
	WM	ns	ns	ns	ns	ns	ns	ns
WT	ΔL	ns	ns	ns	ns	ns	ns	ns
	P99A	ns	ns	ns	ns	ns	ns	ns
	EE	ns	ns	ns	ns	ns	ns	ns
	WM	ns	ns	ns	ns	ns	ns	ns
ΔL	P99A	**	ns	ns	ns	ns	ns	ns
	EE	ns	ns	ns	ns	ns	ns	ns
	WM	*	*	ns	ns	*	ns	ns
P99A	EE	ns	ns	ns	ns	ns	ns	ns
	WM	ns	ns	ns	ns	ns	ns	ns
EE	WM	ns	ns	ns	ns	ns	ns	ns

Supplementary Table 2 Results of one-way ANOVA post-hoc Tukey test for AF647-NT-Lys domain density distribution related to Supplementary Fig. 10c. First two columns indicate combinations tested statistically. The third and the succeeding columns indicate the results of the test in each bin of Supplementary Fig. 10c. *, **, and *** indicate the significant difference at the significance level of 0.05, 0.01, and 0.005, respectively, in one-way ANOVA post-hoc Tukey test. ns indicates not significant. Detailed statistics are shown in the Source Data.

Tested combination		Diameter [nm]							
		85	128	170	213	340	383	426	>426
WT	ΔL	ns	ns	ns	ns	ns	ns	ns	ns
	P99A	*	*	*	ns	ns	ns	**	**
	EE	*	*	*	*	*	ns	***	***
	WM	*	**	*	*	*	*	***	***
ΔL	P99A	*	*	*	ns	ns	ns	**	**
	EE	*	*	*	*	*	ns	***	***
	WM	**	**	*	*	*	*	***	***
P99A	EE	ns	ns	ns	ns	ns	ns	ns	ns
	WM	ns	ns	ns	ns	ns	ns	ns	ns
EE	WM	ns	ns	ns	ns	ns	ns	ns	ns

Supplementary Table 3 Results of one-way ANOVA post-hoc Tukey test for Gag-mEos4b derivatives domain diameter distribution related to Supplementary Fig. 10d. The first two columns indicate combinations tested statistically. The third and succeeding columns indicate the results of the test in each bin of Supplementary Fig. 10d. *, **, and *** indicate the significant difference at the significance level of 0.05, 0.01, and 0.005, respectively, in one-way ANOVA post-hoc Tukey test. ns indicates not significant. Detailed statistics are shown in the Source Data.

Test combination		Area [nm^2] 2000
Vec	WT	**
	WM	ns
WT	WM	**

Supplementary Table 4. Results of One-way ANOVA post-hoc Tukey test for AF647-D4 domain area related to Supplementary Fig. 11a. The first two columns indicate combinations tested statistically. The third column indicates the results of the test in each bin of Supplementary Fig. 11a. ** indicates a significant difference at the significance level of 0.01 in one-way ANOVA post-hoc Tukey test. ns indicates not significant.

Tested combination		125 /domain
Vec	WT	ns
	WM	*
WT	WM	ns

Supplementary Table 5 Results of one-way ANOVA post-hoc Tukey test for distribution of AF647-D4 localization number per domain related to Supplementary Fig. 11. The first two columns indicate combinations tested statistically. The third column indicates the results of the test in each bin of Supplementary Fig. 11b. * indicates the significant difference at the significance level of 0.05 in one-way ANOVA post-hoc Tukey test. ns indicates not significant.

Tested combination		Density [/nm^2]			
		0.005	0.015	0.025	0.04
vec	WT	*	*	*	**
	WM	ns	ns	ns	ns
WT	WM	ns	ns	ns	**

Supplementary Table 6 Results of one-way ANOVA post-hoc Tukey test for AF647-D4 domain density distribution related to Supplementary Fig. 11c. The first two columns indicate combinations tested statistically. The third and succeeding columns indicate the results of the test in each bin of Supplementary Fig. 11c. * and ** indicate the significant difference at the significance level of 0.05 and 0.01, respectively, in one-way ANOVA post-hoc Tukey test. ns indicates not significant.

Supplementary Note

Validation of the double exponential equation model in the analysis of FRET-FLIM data

The fluorescence decays in the data of Fig. 4 and Fig. 5 were first fitted by a single exponential equation and a double exponential equation. The χ^2 values (mean ± SD) in Fig. 4 were: donor alone, 1.062 ± 0.014 and 1.067 ± 0.014; donor + acceptor, 1.653 ± 0.39 and 1.114 ± 0.052 for the single and double exponential equation, respectively. The χ^2 values in Fig. 5 were: donor alone, 1.081 ± 0.043 and 1.091 ± 0.037; donor + acceptor, 1.075 ± 0.022 and 1.068 ± 0.021 for the single and double exponential equation, respectively. These values and paired two-tailed t-test indicated that, in the presence of acceptor, the double exponential equation better fitted the decay curves than the single exponential equation did (donor + acceptor, $p = 3.1 \times 10^{-9}$ in Fig. 4; $p = 7.0 \times 10^{-5}$ in Fig. 5) and in the absence of acceptor, the single exponential equation fitted better than the double exponential equation (donor alone, $p = 7.0 \times 10^{-4}$ in Fig. 4; $p = 0.025$ in Fig. 5).

Localization uncertainties of fluorescent probes in the super-resolution microscopy

The localization uncertainty on each localization was calculated by default in the analysis of a localization list by ThunderSTORM according to the supplementary note of Ovesny et al.[1] The mean ± SEM of the localization uncertainties, as described below, was calculated from the means of three independent experiments using Gag WT-expressing cells' data. Localization uncertainties 11.29 ± 0.37 nm and 12.48 ± 0.15 nm for AF647-NT-Lys and Gag-mEos4b, 11.19 ± 0.19 nm and 12.04 ± 0.06 nm for AF647-D4 and Gag-mEos4b, and 11.12 ± 0.36 nm and 20.18 ± 0.30 nm for AF647-NT-Lys and JF549-D4, respectively, were obtained.

Kernel density estimation used in flimDiagRam

Kernel density estimation (KDE) is a non-parametric technique to estimate density of scatter points and make a smooth empirical probability density estimate function. KDE allows to infer the underlying probability function even at points where data do not exist. The bivariate kernel function is the follow:

$$\hat{f}(x,y) = \frac{1}{nh_x h_y} \sum_{i=1}^{n} K\left(\frac{x_i - x}{h_x}, \frac{y_i - y}{h_y}\right) \quad (1)$$

,where $\{x_i, y_i\}$, $i = 1, 2, \ldots$, n, is a bin in 2D plot, and h_x and h_y ($h > 0$) are smoothing coefficients (bandwidths) that control the extent of smoothing. In the flimDiagRam (https://github.com/jgodet/flimDiagRam), $K(x, y)$ used a bivariate Gaussian kernel.

Supplementary Reference

1. Ovesny, M., Krizek, P., Borkovec, J., Svindrych, Z. & Hagen G. M. ThunderSTORM: a comprehensive ImageJ plug-in for PALM and STORM data analysis and super-resolution imaging. *Bioinformatics* **30**, 2389-2390 (2014).

Fig. 1 Unprocessed images of TLC

Fig. 1a

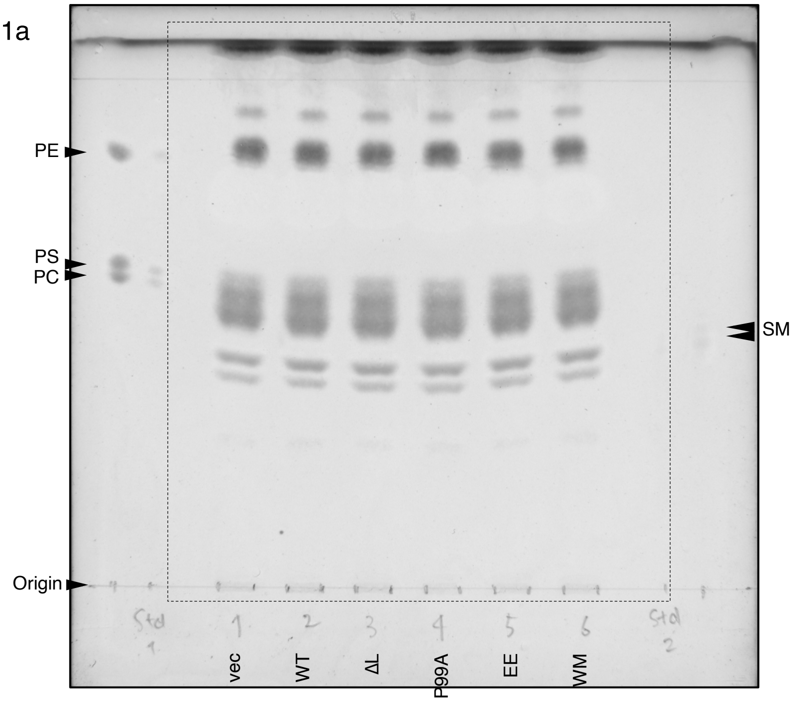

Fig. 1c

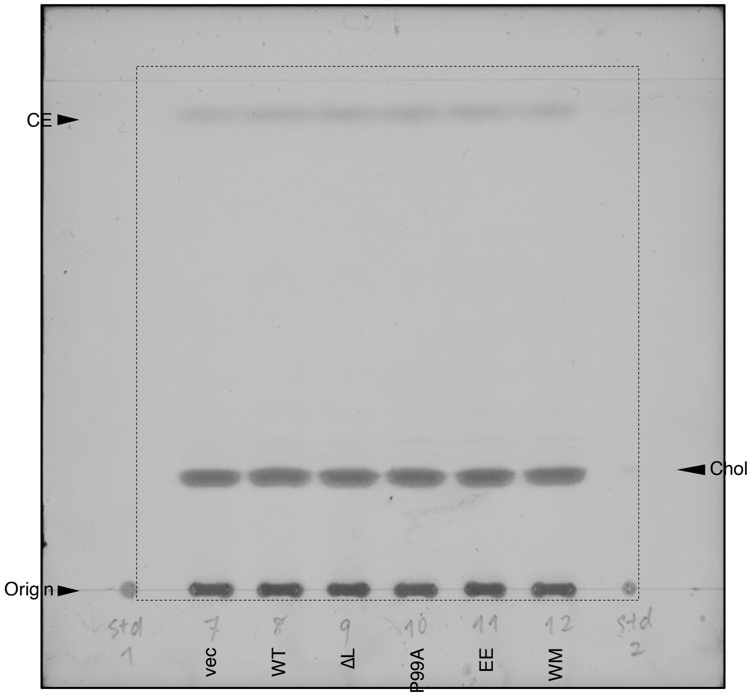

Black rectangles indicate cropped positions in the TLCs.

Supplementary Fig. 2 Unprocessed images of Western blots

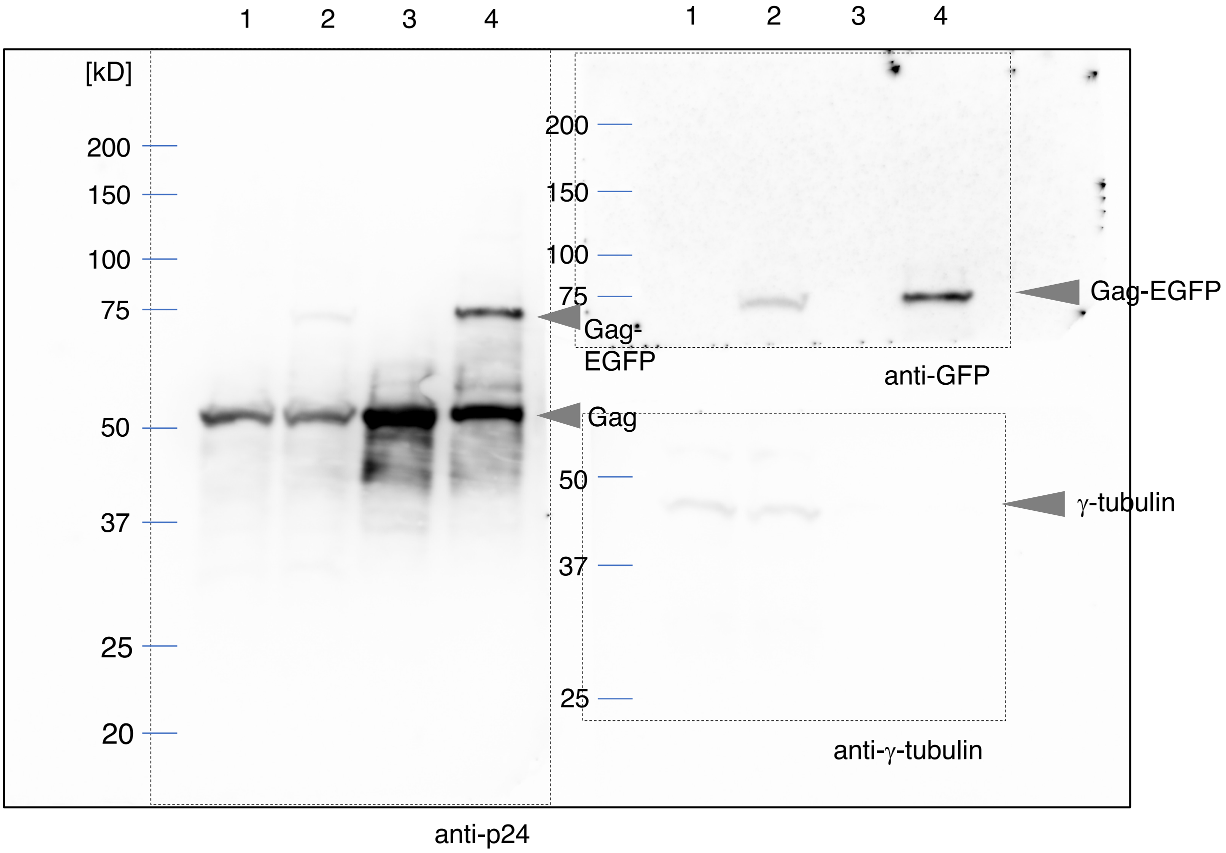

One membrane was cut into three pieces so that proteins of interest were detected. Black dashed lines indicate cropped positions in the membrane.

Supplementary Fig. 4 Unprocessed images of Western blots

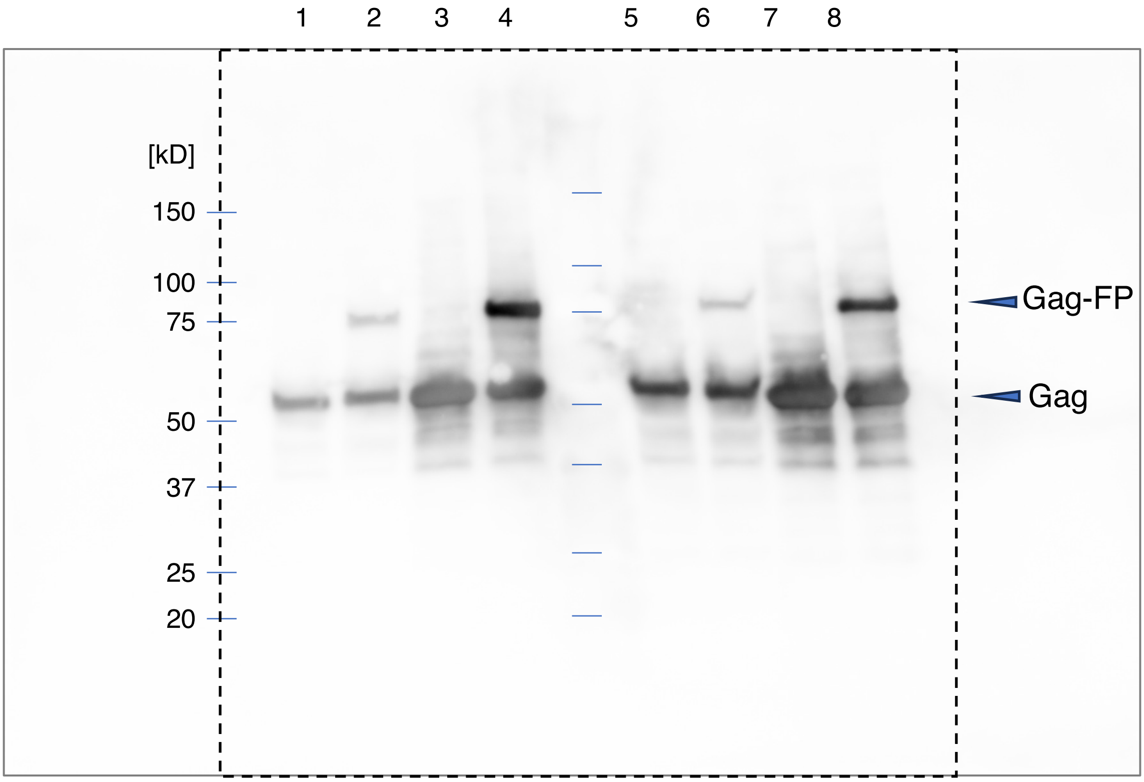

anti-HIV-1 p24

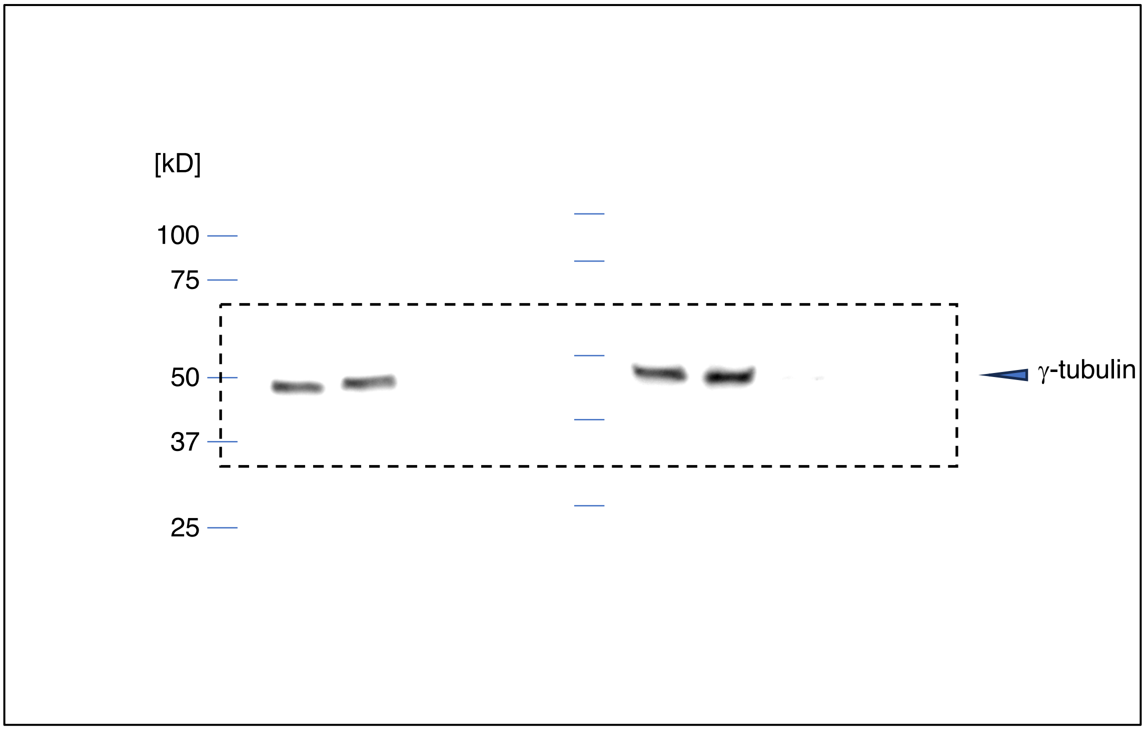

anti-γ-tubulin

Black dashed lines indicate cropped positions in the membrane.

Supplementary Fig. 5 Unprocessed images of Western blots

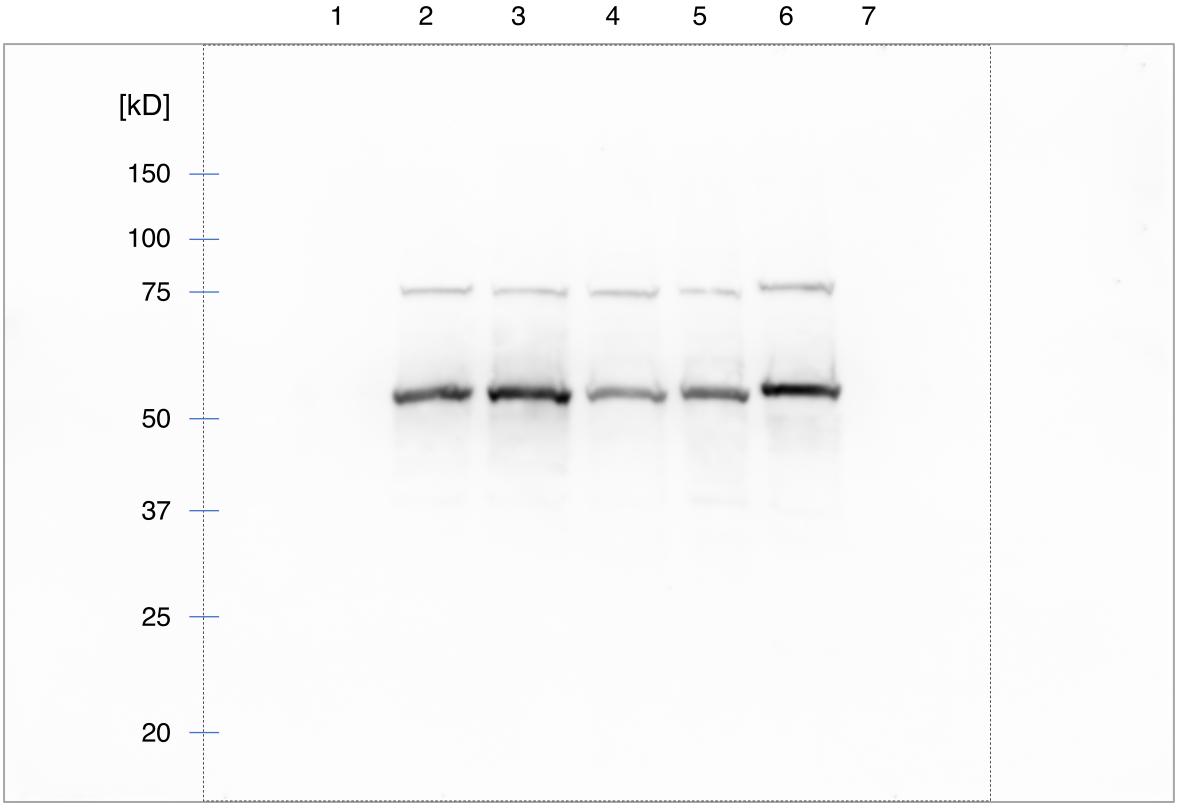

anti-p24

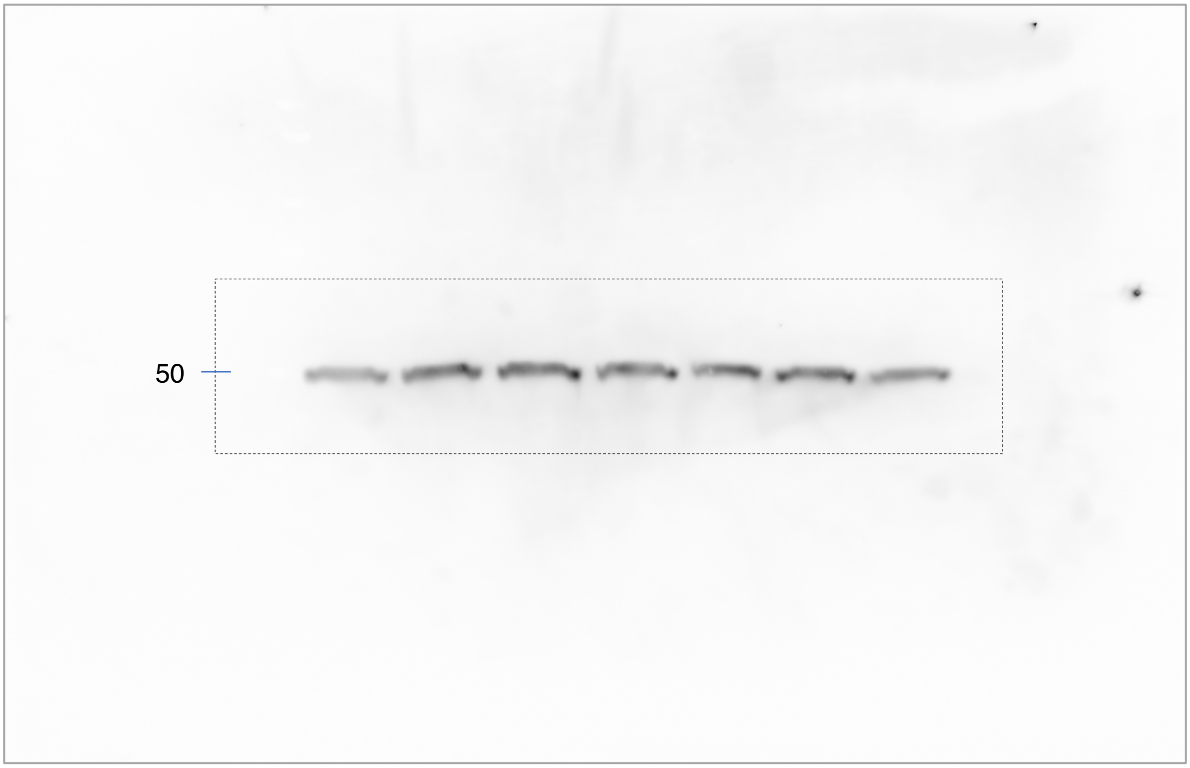

anti-α-tubulin

Black dashed lines indicate cropped positions in the membranes. Both images were obtained from the same membrane that was reprobed with different antibodies.

Supplementary Fig. 6 Unprocessed images of Western blots

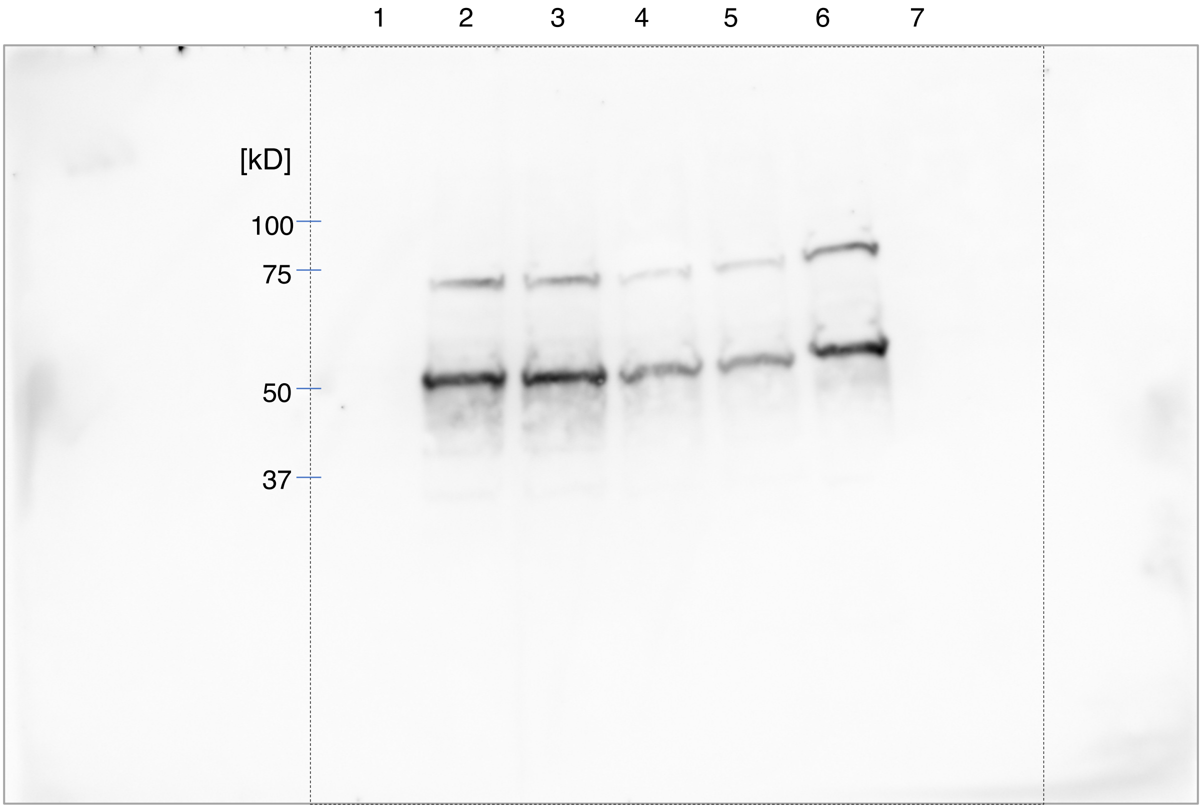

anti-p24

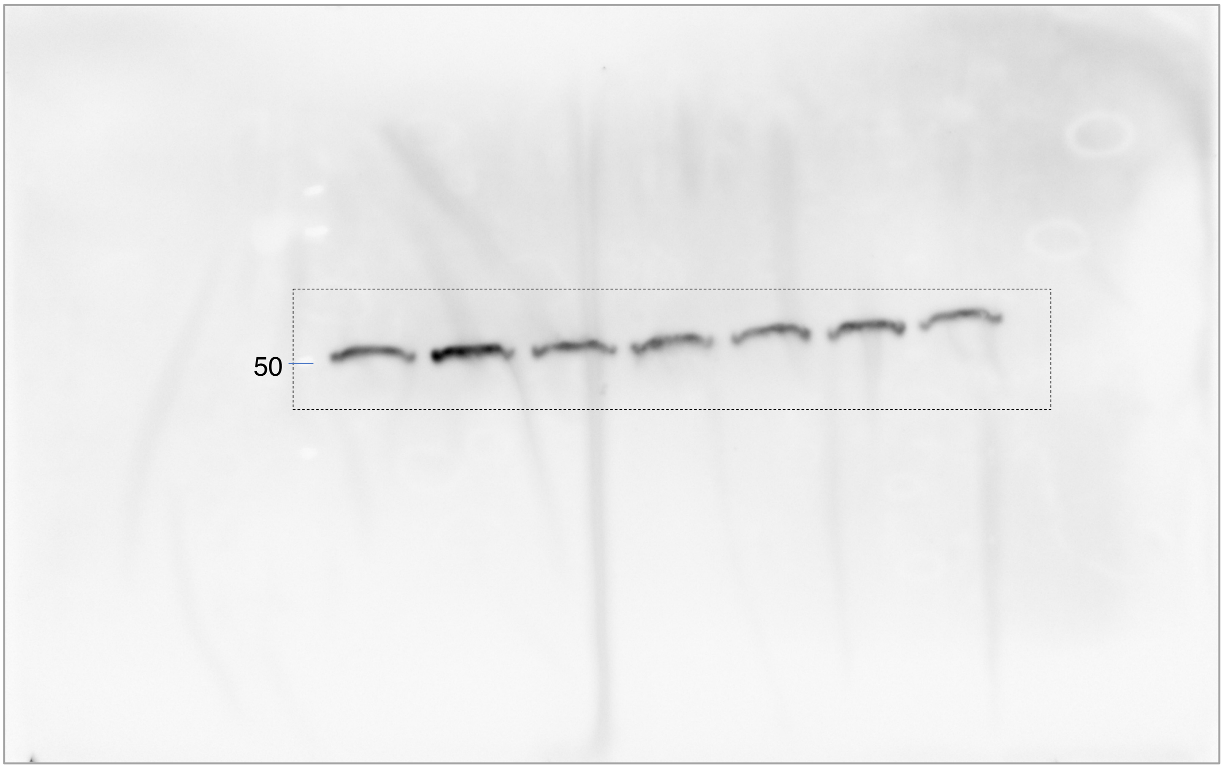

anti-α-tubulin

Black dashed lines indicate cropped positions in the membranes. Both images were obtained from the same membrane that was reprobed with different antibodies.
