# Supplementary material for: HIV-1 Gag targeting to the plasma membrane reorganizes sphingomyelin-rich and cholesterol-rich lipid domains
Source: Nat Commun. 2023 Nov 21;14:7353. doi: 10.1038/s41467-023-42994-w (PMC10663554; doi:10.1038/s41467-023-42994-w)
Supplement: Supplementary file 3 — Reporting Summary [file 41467_2023_42994_MOESM3_ESM.pdf]

nature portfolio

Corresponding author(s): Nario Tomishige
Yves Mély
Toshihide Kobayashi

Last updated by author(s): Oct 20, 2023

Reporting Summary

Nature Portfolio wishes to improve the reproducibility of the work that we publish. This form provides structure for consistency and transparency in reporting. For further information on Nature Portfolio policies, see our Editorial Policies and the Editorial Policy Checklist.

Statistics

For all statistical analyses, confirm that the following items are present in the figure legend, table legend, main text, or Methods section.

n/a	Confirmed	
☐	☒	The exact sample size (*n*) for each experimental group/condition, given as a discrete number and unit of measurement
☐	☒	A statement on whether measurements were taken from distinct samples or whether the same sample was measured repeatedly
☐	☒	The statistical test(s) used AND whether they are one- or two-sided<br>*Only common tests should be described solely by name; describe more complex techniques in the Methods section.*
☒	☐	A description of all covariates tested
☒	☐	A description of any assumptions or corrections, such as tests of normality and adjustment for multiple comparisons
☐	☒	A full description of the statistical parameters including central tendency (e.g. means) or other basic estimates (e.g. regression coefficient) AND variation (e.g. standard deviation) or associated estimates of uncertainty (e.g. confidence intervals)
☐	☒	For null hypothesis testing, the test statistic (e.g. *F*, *t*, *r*) with confidence intervals, effect sizes, degrees of freedom and *P* value noted<br>*Give P values as exact values whenever suitable.*
☒	☐	For Bayesian analysis, information on the choice of priors and Markov chain Monte Carlo settings
☒	☐	For hierarchical and complex designs, identification of the appropriate level for tests and full reporting of outcomes
☐	☒	Estimates of effect sizes (e.g. Cohen's *d*, Pearson's *r*), indicating how they were calculated

Our web collection on statistics for biologists contains articles on many of the points above.

Software and code

Policy information about availability of computer code

Data collection	µManager ver. 1.4.22 was used for PALM/dSTORM imaging, ZEN2008 (Carl Zeiss) for FRAP and confocal imaging, FV10-ASW4.2 (Olympus) for confocal imaging, SPCM ver. 9.83 (Becker & Hickl) for FLIM-FRET, Image Reader LAS-4000 ver. 1.0 (GE healthcare) for imaging of TLC plates and Western blotting membranes.
Data analysis	DoM plugin (ver. 1.1.6) and ThunderSTORM plugin (ver. 1.3) for Fiji (ver. 2.14.0/1.54f), and PoCA (ver. 0.5.0) (https://github.com/flevet/PoCA) were used for PALM/dSTORM data analysis, easyFRAP (standalone version; http://ccl.med.upatras.gr/tools-easyfrap/) and MATLAB (2023a) for FRAP data analysis, SPCImage ver. 8.1 (Becker & Hickl) and flimDiagRam (https://github.com/jgodet/flimDiagRam) for FLIM-FRET data analysis, Multi Gauge ver. 3.1 (GE healthcare) for TLC plate analysis, and Microsoft Excel (for Mac, ver. 16) and GraphPad Prism ver. 5.04 for all the data analyses.

For manuscripts utilizing custom algorithms or software that are central to the research but not yet described in published literature, software must be made available to editors and reviewers. We strongly encourage code deposition in a community repository (e.g. GitHub). See the Nature Portfolio guidelines for submitting code & software for further information.

Data

Policy information about availability of data

All manuscripts must include a data availability statement. This statement should provide the following information, where applicable:

- Accession codes, unique identifiers, or web links for publicly available datasets
- A description of any restrictions on data availability
- For clinical datasets or third party data, please ensure that the statement adheres to our policy

All data generated in this study have shown in the Figures, Supplementar Information, and Source Data file. The Source Data file is provided with this paper and also available in the data repositery figshare (DOI: 10.6084/m9.figshare.24290941).

Research involving human participants, their data, or biological material

Policy information about studies with human participants or human data. See also policy information about sex, gender (identity/presentation), and sexual orientation and race, ethnicity and racism.

Reporting on sex and gender	n/a
Reporting on race, ethnicity, or other socially relevant groupings	n/a
Population characteristics	n/a
Recruitment	n/a
Ethics oversight	n/a

Note that full information on the approval of the study protocol must also be provided in the manuscript.

Field-specific reporting

Please select the one below that is the best fit for your research. If you are not sure, read the appropriate sections before making your selection.

[x] Life sciences [ ] Behavioural & social sciences [ ] Ecological, evolutionary & environmental sciences

For a reference copy of the document with all sections, see nature.com/documents/nr-reporting-summary-flat.pdf

Life sciences study design

All studies must disclose on these points even when the disclosure is negative.

Sample size	The sample sizes in a single experiment were empirically determined according to the time available to perform image acquisitions for all samples on the same day.
Data exclusions	During processing raw data in PALM/dSTORM and FLIM-FRET measurements, data were filtered by sigma (120 nm < sigma < 180 nm, intensity > 100, and chi2 > 0.5 or > 0.2) for PLAM/dSTORM analysis and by alpha1 (1 < alpha1 < 99) and tau1 (not equal to 20.0) for FLIM-FRET analysis to remove unrealistic values. For FRAP experiments, samples with an insufficient bleaching depth (< 40%) and with cell movement during acquisition were discarded.
Replication	All the quantitative microscopy experiments and lipid quantification were repeated independently three times. In PALM/dSTORM, FRAP, and FLIM-FRET, 4-6 cells, 7-11 cells, and 9 or 10 cells, respectively, were analyzed in each experiment (see these details below). In all these results, a null hypothesis was tested using paired two-tailed t-test or repeated measures one-way ANOVA post-hoc Tukey test. In Western blotting, the experiments were repeated twice and the results were further confirmed by using the same constructs with different fluorophores. The detailed sample numbers over three experiments are following: in PALM/dSTORM, n = 16 (P99A, EE, and WM), 17 (vec and ΔL), and 18 (WT) for AF647-NT-Lys and Gag-mEos4b combination, n = 15 (vec), 16 (WT), and 16 (WM) for AF647-D4 and Gag-mEos4b combination, and n = 18 (vec) and 15 (WT) for AF647-NT-Lys and JF549-D4 combination; in FRAP, n = 29 (vec) and 28 (Gag) for EGFP-NT-Lys, and 30 (vec), and 25 (Gag) for EGFP-D4; in FLIM-FRET, n = 30 (vec, WT, and WM) and 29 (DA) for EGFP-NT-Lys and mCherry-NT-Lys combination, and n = 30 (vec, DA, WT, ΔL, P99A, and WM) and 29 (EE) for EGFP-NT-Lys and mCherry-D4 combination. All attempts at replication were successful.
Randomization	Samples were allocated randomly.
Blinding	In our experiments, blinding was not performed and cells to be imaged were randomly chosen. The intended selection of cells to be imaged was not possible because the results in our experiments were obtained only after analyzing the acquired image data.

Reporting for specific materials, systems and methods

We require information from authors about some types of materials, experimental systems and methods used in many studies. Here, indicate whether each material, system or method listed is relevant to your study. If you are not sure if a list item applies to your research, read the appropriate section before selecting a response.

Materials & experimental systems

n/a	Involved in the study
☐	☒ Antibodies
☐	☒ Eukaryotic cell lines
☒	☐ Palaeontology and archaeology
☒	☐ Animals and other organisms
☒	☐ Clinical data
☒	☐ Dual use research of concern
☒	☐ Plants

Methods

n/a	Involved in the study
☒	☐ ChIP-seq
☒	☐ Flow cytometry
☒	☐ MRI-based neuroimaging

Antibodies

Antibodies used

Anti-HIV-1 p24 (ATCC, Cat# ARP-3537, clone 183-H12-5C, Lot#130149, dilution rate x 5,000), anti-GFP (Invitrogen, Cat#G10362, x 3,000), anti-gamma-tubulin (Sigma-Aldrich, cat# T-5326, clone GTU-88, x 5,000), or anti-alpha-tubulin (Sigma-Aldrich, Cat# T-9026, clone DM1A, x 2,000) were used for primary antibody. Anti-mouse IgG horseradish peroxidase (HRP) conjugated (GE healthcare, Cat# NA931V, Lot# 17062552, x 5,000) or anti-rabbit IgG HRP conjugated (GE healthcare, Cat# NA934, Lot# 17853438, x 5,000) for secondary antibody.

Validation

Anti-HIV-1 p24, distributor website (https://www.hivreagentprogram.org/Catalog/HRPMonoclonalAntibodies/ARP-3537.aspx); anti-GFP, manufacturer's website (https://www.thermofisher.com/antibody/product/GFP-Antibody-Recombinant-Monoclonal/G10362); anti-gamma-tubulin, manufacturer's website (https://www.sigmaaldrich.com/FR/fr/product/sigma/t5326); anti-alpha-tubulin, manufacturer's website (https://www.sigmaaldrich.com/FR/fr/product/sigma/t9026).

Eukaryotic cell lines

Policy information about cell lines and Sex and Gender in Research

Cell line source(s)

HeLa cells were from ATCC (CCL-2).

Authentication

HeLa cell line used in this study was not authenticated.

Mycoplasma contamination

HeLa cells were confirmed tested negative for mycoplasma contamination.

Commonly misidentified lines
(See ICLAC register)

No commonly misidentified cell lines were used in this study.
